# Deletion of tumour necrosis factor α receptor 1 elicits an increased TH17 immune response in the chronically inflamed liver

**DOI:** 10.1038/s41598-019-40324-z

**Published:** 2019-03-12

**Authors:** Laura Berkhout, Roja Barikbin, Birgit Schiller, Gevitha Ravichandran, Till Krech, Katrin Neumann, Gabriele Sass, Gisa Tiegs

**Affiliations:** 1 Institute of Experimental Immunology and Hepatology, University Medical Center Hamburg-Eppendorf, Hamburg, Germany; 2 Institute of Pathology, University Medical Center Hamburg-Eppendorf, Hamburg, Germany; 3 California Institute for Medical Research, San Jose, CA USA

## Abstract

Tumour necrosis factor α receptor 1 (TNFR1) activation is known to induce cell death, inflammation, and fibrosis but also hepatocyte survival and regeneration. The multidrug resistance protein 2 knockout (*Mdr2*^−*/*^) mice are a model for chronic hepatitis and inflammation-associated hepatocellular carcinoma (HCC) development. This study analysed how the absence of TNFR1 mediated signalling shapes cytokine and chemokine production, immune cell recruitment and ultimately influences liver injury and fibrotic tissue remodelling in the *Mdr2*^*−/−*^ mouse model. We show that *Tnfr*1^*−/−*^*/Mdr2*^*−/−*^ mice displayed increased plasma levels of ALT, ALP, and bilirubin as well as a significantly higher collagen content, and markers of fibrosis than *Mdr2*^*−/−*^ mice. The expression profile of inflammatory cytokines (*Il1b, Il23, Tgfb1, Il17a*), chemokines (*Ccl2, Cxcl1, Cx3cl1*) and chemokine receptors (*Ccr6, Cxcr6, Cx3cr1*) in livers of *Tnfr1*^*−/−*^*/Mdr2*^*−/−*^ mice indicated TH17 cell infiltration. Flow cytometric analysis confirmed that the aggravated tissue injury in *Tnfr1*^*−/−*^/*Mdr2*^*−/−*^ mice strongly correlated with increased hepatic recruitment of TH17 cells and enhanced IL-17 production in the injured liver. Moreover, we observed increased hepatic activation of RIPK3 in *Tnfr1*^*−/−*^*/Mdr2*^*−/−*^ mice, which was not related to necroptotic cell death. Rather, frequencies of infiltrating CX3CR1^+^ monocytes increased over time in livers of *Tnfr1*^*−/−*^*/Mdr2*^*−/−*^ mice, which expressed significantly higher levels of *Ripk3* than those of *Mdr2*^*−/−*^ mice. Overall, we conclude that the absence of TNFR1-mediated signalling did not improve the pathological phenotype of *Mdr2*^*−/−*^ mice. It instead caused enhanced infiltration of TH17 cells and CX3CR1^+^ monocytes into the injured tissue, which was accompanied by increased RIPK3 activation and IL-17 production.

## Introduction

Chronic liver disease (CLD) is a major global health burden and cause of 2% (>1*10^6^ annually) of all deaths worldwide (2010)^1^. In addition, CLD is often the basis for equally lethal secondary pathologies including pulmonary and cardiac manifestations, hepatorenal syndrome and most prominently, hepatocellular carcinoma (HCC)^2–4^. CLD progresses through distinct phases such as initial injury, subsequent inflammation and fibrotic remodelling which over time culminates in irreversible cirrhosis, mostly independent of the cause^5^. However, the underlying inflammatory and regenerative processes vary, depending on the type of injury and the interplay of cytokines and chemokines with resident as much as recruited immune cell populations, which in turn determine the disease severity and pace of progression. It has been shown that acute and chronic hepatic inflammation, fibrotic tissue remodelling, and potential tumorigenesis is in part promoted by tumour necrosis factor α (TNFα) signalling through TNFα receptor 1 (TNFR1), and to a lesser degree through activation of TNFR2^6,7^ The signalling pathways of both TNF receptors have considerable overlap, with TNFR1 being expressed ubiquitously and responsible for most of the pro-inflammatory, cytotoxic and apoptotic effects of TNFα, while TNFR2 is primarily found on the hematopoietic cell compartment and lacks the intracellular death domain which induces TNFR1-dependent cell death^8^.

Numerous agents targeting TNFα signalling are currently used for treating patients with a variety of inflammatory pathologies^9^. While anti-TNFα therapy is considered to be relatively safe, it still renders patients partially immunosuppressed and consequently more susceptible to secondary infections and possibly cancer due to impaired anti-tumour immunity^10^. Thus, it has been implied, that targeting TNFR1 signalling exclusively, while upholding some of the physiological functions of TNFα signalling through TNFR2, would be a more comprehensive therapeutic approach^11,12^. In order to investigate the distinct effects of TNFR1 signalling during chronic inflammation, we crossbred multidrug resistance protein 2 knockout (*Mdr2*^*−/−*^) mice with *Tnfr1*^*−/−*^ mice, creating double knockout *Tnfr1*^*−/−*^/*Mdr2*^*−/−*^ mice. The *Mdr2* gene encodes a P-glycoprotein which transports phosphatidylcholine into the bile. In the absence of phosphatidylcholine primary bile salts have increased detergent activity, damaging the membranes of surrounding hepatocytes^13^. This constant damage leads to the production of pro-inflammatory cytokines, including TNFα, immune cell infiltration into the injured liver, and progressive fibrotic tissue remodelling^14,15^.

We previously demonstrated that TNFR1 expression depends on the extent of inflammation in the *Mdr2*^*−/−*^model^16^. Loss or inhibition of TNFR1 has previously been shown to be protective in mouse models of acute liver injury^17,18^. In contrast, TNFR1 is also known to induce cytoprotective processes, and the complete absence of TNFR1 signalling reduces the hepatic regenerative capacity and pro-survival signalling through diminished activation of NFκB in the injured liver^19–21^. While during tissue injury, an accurate regenerative response is essential to restore tissue integrity, reduced proliferation in a setting of chronic inflammation might prevent tumour development. We established the *Tnfr1*^*−/−*^/*Mdr2*^*−/−*^ mouse model in order to determine how TNFR1 shapes the immune response during bile acid-induced CLD and affects overall disease severity and progression.

## Results

### The absence of TNFR1 increases tissue injury and fibrotic remodelling in the *Mdr2*^*−/−*^ mouse model

In order to evaluate how the absence of TNFR1 signalling during chronic liver inflammation influences tissue injury and subsequent fibrosis in der *Mdr2*^*−/−*^ mouse model, we used 12-week-old female mice of the respective genotypes (unless specified otherwise). Female *Mdr2*^*−/−*^ mice exhibit an increased pathological phenotype, allowing for a more detailed analysis of the underlying processes^22^. We chose 12-week-old mice to see both, active inflammation and pronounced fibrosis.

**Figure 1:**
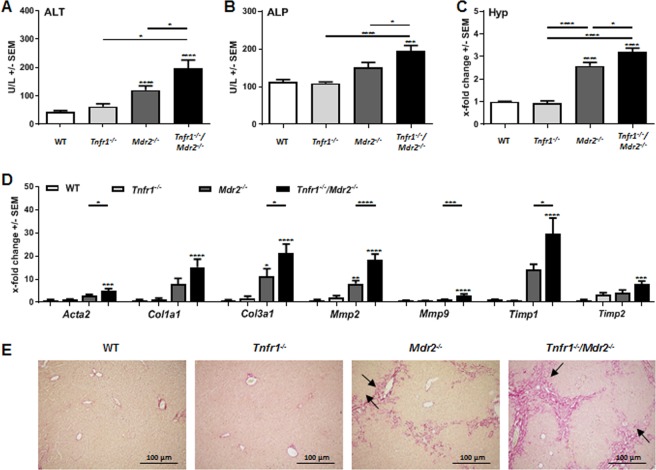
Absence of TNFR1 increased tissue injury in the *Mdr2*^*−/−*^ mouse model. (**A**) ALT and (**B**) ALP levels determined in plasma of WT (n ≥ 4), *Tnfr1*^*−/−*^ (n ≥ 6), *Mdr2*^*−/−*^ (n ≥ 9), and *Tnfr1*^*−/−*^*/Mdr2*^*−/−*^ (n ≥ 9) mice. (**C**) Quantification of the hepatic hydroxyproline content of mice described in A. (**D**) Relative hepatic expression of *Acta2, Col1a1, Col3a1, Mmp2, Mmp9, Timp1*, and *Timp2* of mice described in A, determined by RT-qPCR. (**E**) Representative images (10x) of Sirius Red stained tissue sections of mice described in (**A**). *P ≤ 0.05, ***P ≤ 0.001, ****P ≤ 0.0001.

We observed increased tissue injury in *Tnfr1*^*−/−*^*/Mdr2*^*−/−*^ mice, defined by increased plasma levels of alanine aminotransferase (ALT) and alkaline phosphatase (ALP) (Fig. 1A,B). Both markers for liver injury were increased in *Mdr2*^*−/−*^ mice compared to C57Bl/6 (WT) mice, but still significantly higher in *Tnfr1*^*−/−*^*/Mdr2*^*−/−*^ mice, while *Tnfr1*^*−/−*^ mice showed no increase of either enzyme. Further evidence of increased cholestatic liver injury in *Tnfr1*^*−/−*^*/Mdr2*^*−/−*^ mice were significantly increased plasma levels of bilirubin accompanied with decreased levels of plasma cholesterol in direct comparison to *Mdr2*^*−/−*^ mice (Supplementary Fig. 1A,B).

To assess fibrogenesis, we quantified the hepatic collagen content by measuring hydroxyproline in liver tissue samples of the respective genotypes (Fig. 1C). While the hydroxyproline contents of *Tnfr1*^*−/−*^ mice were comparable to WT animals*, Mdr2*^*−/−*^ mice had significantly increased levels of hydroxyproline compared to the WT mice. Interestingly, the hydroxyproline content of *Tnfr1*^*−/−*^*/Mdr2*^*−/−*^ mice was further significantly elevated compared to *Mdr2*^*−/−*^ mice. Collagen deposition is the direct result of fibrotic remodelling in response to prolonged tissue injury. In line with that, we observed significantly increased gene expression of various markers of fibrosis, including genes for α-smooth muscle actin (*Acta2*), collagen type 1 (*Col1a1*) and type 3 (*Col3a1*), matrix metalloproteinases (*Mmp) 2* and *Mmp9* as well as tissue inhibitors of MMPs (*Timp*) *1* and *Timp2* in the livers *of Tnfr1*^*−/−*^*/Mdr2*^*−/−*^ mice compared to *Mdr2*^*−/−*^ mice (Fig. 1D). The representative Sirius Red stained tissue sections presented in Fig. 1E clearly show that WT and *Tnfr1*^*−/−*^ mice displayed healthy liver parenchyma, with red stained collagen deposition restricted to the basement membrane of the vasculature. *Mdr2*^*−/−*^ and *Tnfr1*^*−/−*^*/Mdr2*^*−/−*^ mice showed, increased Sirius Red positive areas with cholestatic features such as ductular reactions around portal tracts. Overall, we found that the absence of TNFR1 had no beneficial effect on disease pathology, but instead enhanced tissue injury and fibrotic remodelling in the *Mdr2*^*−/−*^ mouse model.

### The absence of TNFR1 alters the cytokine and chemokine milieu in the injured liver

**Figure 2:**
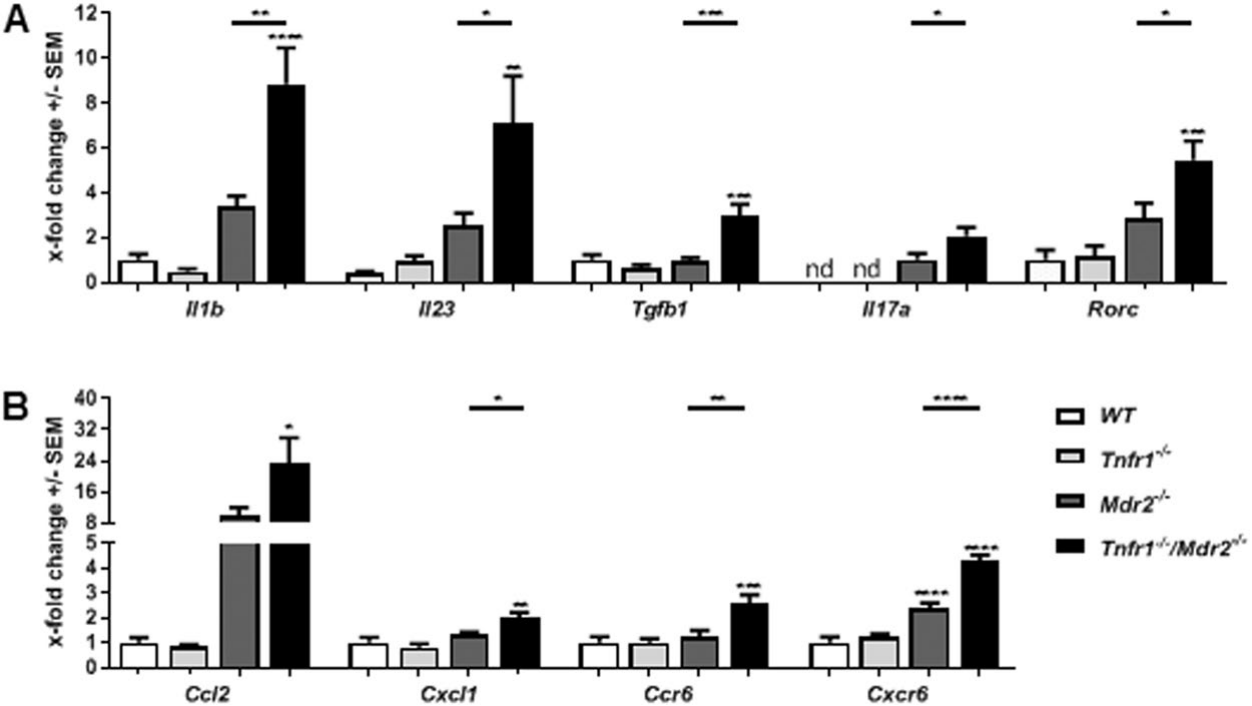
Absence of TNFR1 alters the cytokine and chemokine milieu in the injured liver. Relative hepatic expression levels of (**A**) *Il-1β*, *Il-23*, *Tgfβ1*, *Il-17A*, *Rorγt* and (**B**) *Ccl2*, *Cxcl1*, *Ccr6*, *Cxcr6* of WT (n ≥ 7), *Tnfr1*^*−/−*^ (n ≥ 7), *Mdr2*^*−/−*^ (n ≥ 5), and *Tnfr1*^*−/−*^*/Mdr2*^*−/−*^ (n ≥ 6) mice determined by RT-qPCR. *P ≤ 0.05, **P ≤ 0.01, ***P ≤ 0.001, ****P ≤ 0.0001.

TNFα mediated signalling is essential for several inflammatory pathways, mediating the production and/or release of multiple cytokines and chemokines^23^. We therefore asked whether absence of TNFR1 signalling would affect the cytokine response in the chronically inflamed liver. Figure 2A shows that *Tnfr1*^*−/−*^*/Mdr2*^*−/−*^ mice have significantly increased hepatic gene expression of *Il1b* (*Il-1β*), *Il23* (*Il-23*), *Tgb1* (*Tgfβ1*), and *Il7a* (*Il-17A*) compared to *Mdr2*^*−/−*^ mice. Furthermore, the expression of the gene (*Rorc*) encoding for transcription factor RAR-related orphan receptor gamma t (*RORγt*), was significantly up-regulated in livers of *Tnfr1*^*−/−*^*/Mdr2*^*−/−*^ mice compared to all other genotypes. Furthermore, *Tnfr1*^*−/−*^*/Mdr2*^*−/−*^ mice expressed high levels of the chemokines CC-chemokine ligand 2 (*Ccl2*), showed significantly up-regulated hepatic gene expression of C-X-C motif chemokine ligand 1 (*Cxcl1*), and expressed significantly increased levels of C-C motif chemokine receptor 6 (*Ccr6*) as well as C-X-C motif chemokine receptor 6 (*Cxcr6*) compared to WT, *Tnfr1*^*−/−*^, and *Mdr2*^*−/−*^ mice (Fig. 2B). Overall, the expression analysis showed that the absence of TNFR1 strongly influenced the cytokine and chemokine milieu in the chronically inflamed liver of the *Mdr2*^*−/−*^ background. Considering the cytokine profile presented above including *Il-17A* as well as increased *Rorγt* expression indicating the presence of TH17 cells in the liver of *Tnfr1*^*−/−*^*/Mdr2*^*−/−*^ mice, we decided to further analyse accumulation of TH17 cells in the liver.

### The absence of TNFR1 signalling leads to increased infiltration of TH17 cells into the injured liver

**Figure 3:**
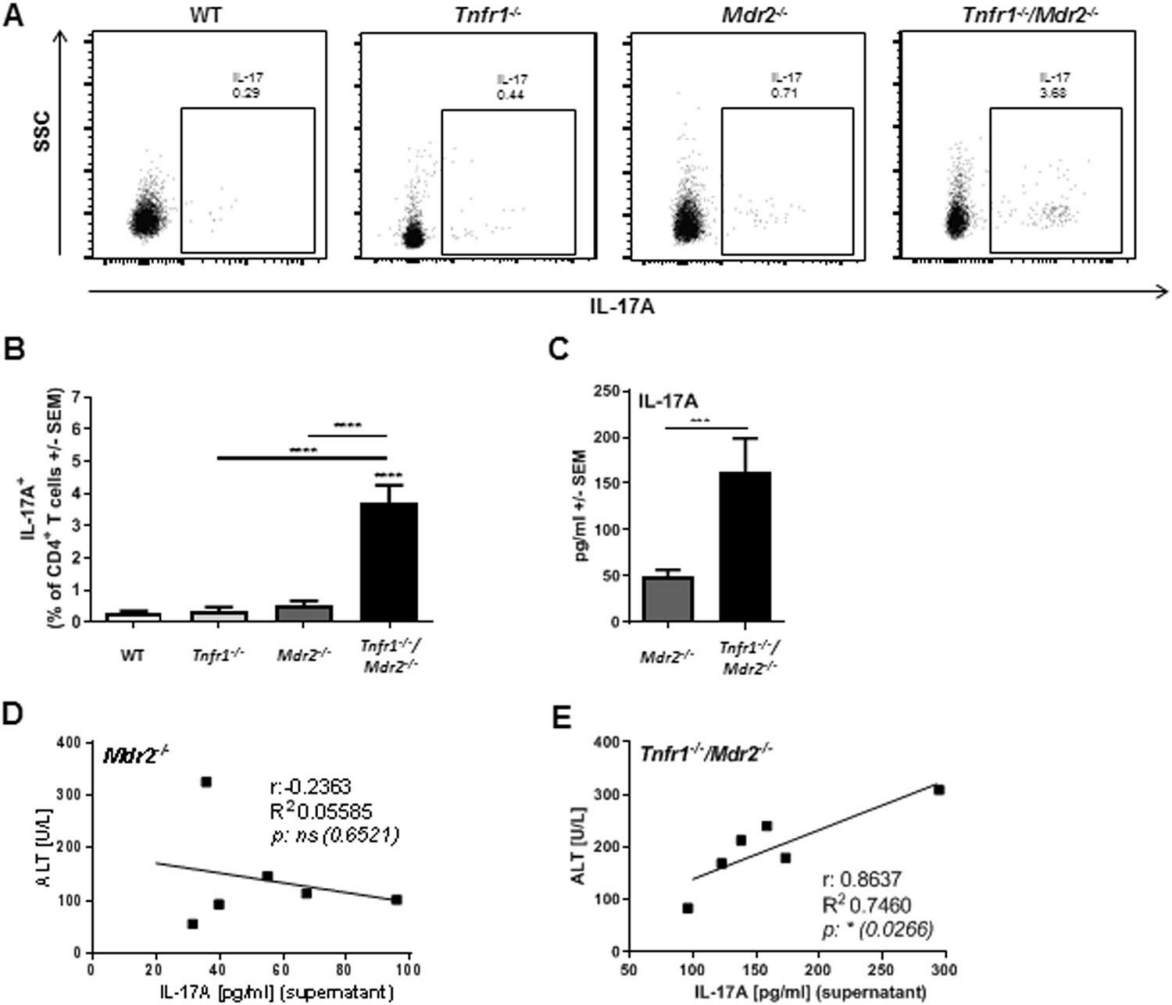
Absence of TNFR1 signalling leads to increased infiltration of TH17 cells into the injured liver. (**A**) Representative dot plots and (**B**) quantification of flow cytometric analysis of TCRβ^+^CD4^+^IL17^+^ TH17 cell populations in the livers of WT (n ≥ 5), *Tnfr1*^*−/−*^ (n ≥ 6), *Mdr2*^*−/−*^ (n ≥ 6), and *Tnfr1*^*−/−*^*/Mdr2*^*−/−*^ (n ≥ 5) mice determined by flow cytometry. (**C**) Concentration of IL-17 in the supernatant of NPCs re-stimulated with PMA & ionomycin for 4 h of mice described in (**A**). (**D**) Correlation between IL-17 production of re-stimulated NPCs with plasma ALT levels of *Mdr2*^*−/−*^ and (**E**) *Tnfr1*^*−/−*^/*Mdr2*^*−/−*^ mice. r: correlation coefficient, R^2^: coefficient of determination. *P ≤ 0.05, **P ≤ 0.01, ***P ≤ 0.001, ****P ≤ 0.0001.

In line with the observation that the absence of TNFR1 leads to an altered microenvironment rich in cytokines and chemokines known to be involved in the recruitment and activation of TH17 cells, flow cytometric analysis revealed an increased frequency of IL-17A-expressing TH17 cells in the livers of *Tnfr1*^*−/−*^*/Mdr2*^*−/−*^ mice (Fig. 3A,B, gating strategy in Supplementary Fig. 2A). For WT, *Tnfr1*^*−/−*^, and *Mdr2*^*−/−*^ mice, we observed only negligible frequencies of hepatic TH17 cells. Furthermore, *ex vivo* stimulation of liver derived non-parenchymal cells (NPCs) with phorbol 12-myristate 13-acetate (PMA) & ionomycin revealed that hepatic immune cells derived from *Tnfr1*^*−/−*^*/Mdr2*^*−/−*^ mice produced significantly more IL-17A than hepatic NPCs from *Mdr2*^*−/−*^ mice (Fig. 3C). While *ex vivo* IL-17A production by *Mdr2*^*−/−*^ NPCs was not associated with the degree of tissue injury, as defined by plasma levels of ALT (Fig. 3D), it was apparent that production of IL-17A in livers of *Tnfr1*^*−/−*^*/Mdr2*^*−/−*^ mice, was directly correlated with the extent of tissue damage (Fig. 3E).

TNFR1 ablation has been shown to diminish the regenerative capacity of the liver due to reduced NFκB activity, which leading to reduced levels of IL-6, and consequently to insufficient STAT3 activation^20,21^. However, IL-17 is also known to be involved in the onset of regeneration by inducing the production of IL-6 and IL-22, both potent inducers of STAT3^24^. In line with that, Legendplex analysis revealed robust IL-6 concentrations in the plasma of *Tnfr1*^*−/−*^*/Mdr2*^*−/−*^ mice and significantly higher plasma levels of IL-22 compared to *Mdr2*^*−/−*^ mice (Supplementary Fig. 3A). In line with that, several genes encoding for proliferation markers including proliferating cell nuclear antigen (PCNA), Cyclin A2 (CCNA2) and cyclin-dependent kinase 1 (CDK1) were significantly increased in livers of *Tnfr1*^*−/−*^*/Mdr2*^*−/−*^ mice (Supplementary Fig. 3B). Since *Mdr2*^*−/−*^ mice are a mouse model of inflammation induced tumour development, and IL-17 has been closely associated with strong induction of regeneration and angiogenesis in the tumour microenvironment, we analysed the gene expression of known HCC tumour markers in *Tnfr1*^*−/−*^*/Mdr2*^*−/−*^ mice^25,26^. We observed up-regulated gene expression of tumour markers including tumour necrosis factor α induced protein (*Tnfaip*; A20), secreted phospho protein 1 (*Ssp1*, OPN) and α-feto protein (*Afp*) (Supplementary Fig. 3C) in 12-week-old *Tnfr1*^*−/−*^*/Mdr2*^*−/−*^ mice^27,28^. Overall, we observed that TH17 cells and their signature cytokines are increased in the injured livers of *Tnfr1*^*−/−*^*/Mdr2*^*−/−*^ mice (Fig. 3), while hepatic gene expression of markers of regeneration and possibly tumour development appeared to be rather activated rather than impaired in the absence of TNFR1 (Supplementary Fig. 3).

### The absence of TNFR1 leads to necroptosis-independent activation of RIPK3 in the chronically inflamed liver

**Figure 4:**
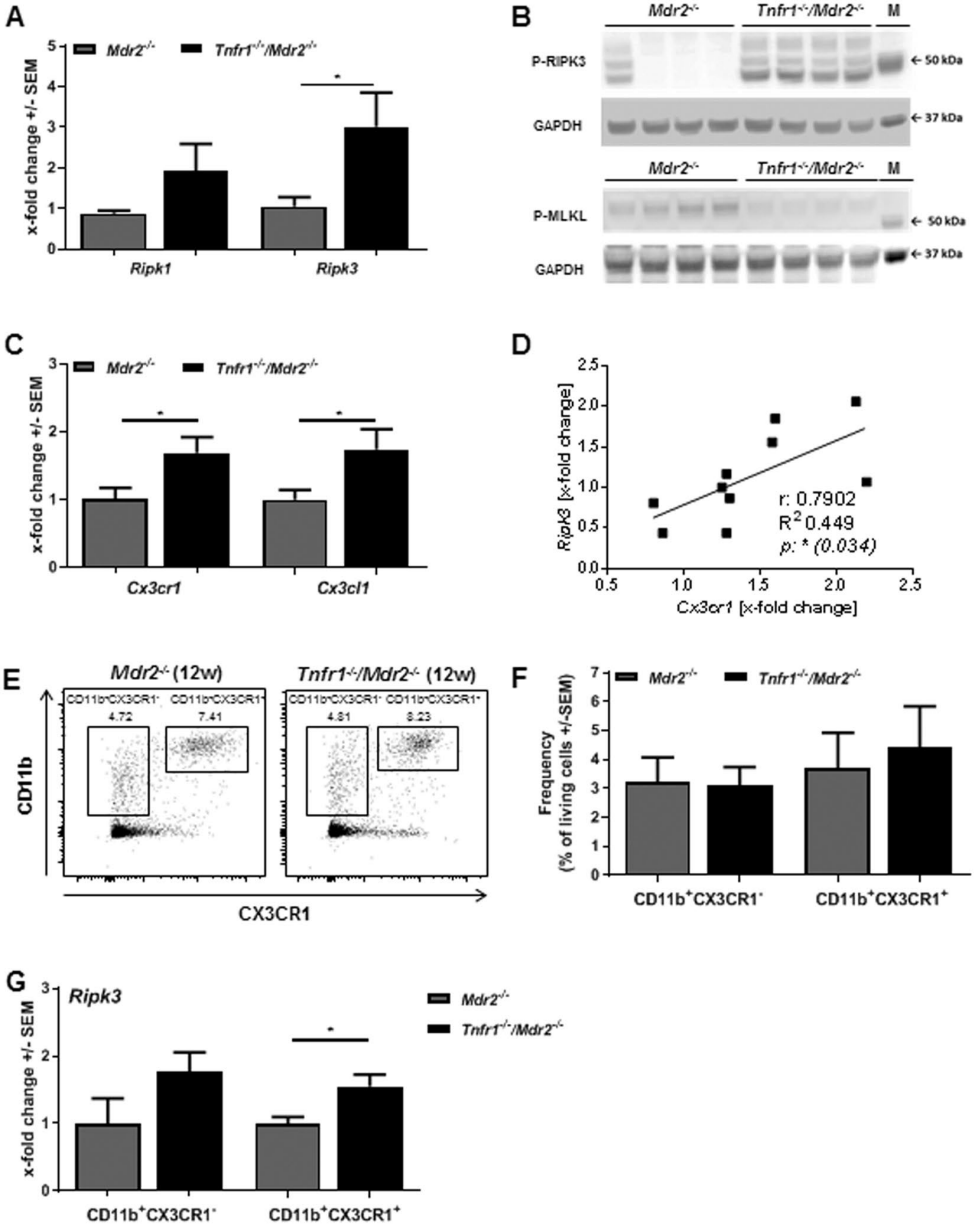
Absence of TNFR1 leads to necroptosis independent activation of RIPK3 and CX3CR1^+^ monocyte recruitment into the chronically inflamed liver. (**A**) Relative hepatic expression levels of *Ripk1*, *Ripk3* determined by RT-qPCR in tissue samples of *Mdr2*^*−/−*^ (n ≥ 3), and *Tnfr1*^*−/−*^/*Mdr2*^*−/−*^ (n ≥ 4) mice. Western blot of (**B**) phosphorylated RIPK3 (P-RIPK3) and MLKL (P-MLKL) with respective GAPDH as loading control in livers of mice described in A. Each line depicts one animal. The samples for the P-RIPK3 and P-MLKL WBs were derived from the same experiment and gels/blots were processed in parallel. Images of the full length blots are presented in Supplementary Fig. 4. (**C**) Relative hepatic expression levels of *Cx3cr1* and *Cx3cl1* determined by RT-qPCR in liver tissue samples of mice described in (**A**). (**D**) Correlation of hepatic expression levels of *Ripk3* with *Cx3cr1* of *Tnfr1*^*−/−*^/*Mdr2*^*−/−*^ mice. (**E**) Representative dot plots and (**F**) quantification of flow cytometric analysis of CD11b^+^CX3CR1^+^ and CD11b^+^CX3CR1^-^ cell populations in the livers of mice described in (**A**). (**G**) Relative expression of *Ripk3* in CD11b^+^ and CD11b^+^CX3CR1^+^ cells of *Mdr2*^*−/−*^ and *Tnfr1*^*−/−*^/*Mdr2*^*−/−*^ mice determined by RT-qPCR. M: kDa marker, r: correlation coefficient, R^2^: coefficient of determination. *P ≤ 0.05, **P ≤ 0.01.

TNFR1 contains an intracellular death domain which can induce several forms of cell death including apoptosis and necroptosis^23^. Therefore, reduced cell death in the livers of *Tnfr1*^*−/−*^*/Mdr2*^*−/−*^ compared to *Mdr2*^*−/−*^ mice had to be expected. As our data indicated the opposite effect, we analysed apoptotic cell death by measurement of activated caspase-3 (western blot), but failed to observe significant differences between *Mdr2*^*−/−*^ and *Tnfr1*^*−/−*^*/Mdr2*^*−/−*^ mice (data not shown). We investigated gene expression levels of the known mediators of necroptosis, namely receptor interacting protein kinase 1 (*Ripk1*) and 3 (*Ripk3*). While *Ripk1* was slightly elevated in *Tnfr1*^*−/−*^*/Mdr2*^*−/−*^ mice, a significant increase of hepatic *Ripk3* expression was observed in *Tnfr1*^*−/−*^*/Mdr2*^*−/−*^ mice compared to *Mdr2*^*−/−*^ mice (Fig. 4A). Necroptosis is mediated by the necrosome, a cytosolic complex consisting of RIPK1, RIPK3 and the mixed lineage kinase domain like pseudokinase (MLKL). We performed western blot analysis of phosphorylated RIPK3 and MLKL in order to investigate their activation state in the injured liver. We demonstrated increased RIPK3 activation in livers of *Tnfr1*^*−/−*^*/Mdr2*^*−/−*^ mice compared to *Mdr2*^*−/−*^ mice (Fig. 4B). However, the opposite effect was observed for phosphorylated MLKL (Fig. 4B). Since MLKL is indispensable for necroptosis, these findings suggest a necroptosis-independent role of RIPK3 during chronic liver injury in the absence of TNFR1. Morriwaki *et al*. were among the first to describe a necroptosis-independent function of RIPK3 by showing that RIPK3 activity is involved in cytokine production in a CX3CR1^+^ monocytic cell population^29^. Gene expression analysis revealed increased *Cx3cr1* and *Cx3cl1* expression in livers of *Tnfr1*^*−/−*^*/Mdr2*^*−/−*^ mice compared to *Mdr2*^*−/−*^ mice (Fig. 4C). Correlation analysis showed that animals in which hepatic *Ripk3* expression was increased also expressed higher levels of the chemokine receptor *Cx3cr1* (Fig. 4D). In order to further investigate a possible role of RIPK3 activity in the monocytic cell compartment, we sorted hepatic CD11b^+^CX3CR1^+^ as well as CD11b^+^CX3CR1^−^ monocytes and analysed *Ripk3* expression by qRT-PCR. Although no differences in the hepatic frequencies of both cell populations were observed in 12-week-old *Tnfr1*^*−/−*^*/Mdr2*^*−/−*^ mice and *Mdr2*^*−/−*^ mice (Fig. 4E,F, gating strategy in Supplementary Fig. 2B), we detected significantly increased expression of *Ripk3* in CD11b^+^CX3CR1^+^ monocytes derived from livers of *Tnfr1*^*−/−*^*/Mdr2*^*−/−*^ compared to those derived from *Mdr2*^*−/−*^ mice (Fig. 4G). These results implicate that the absence of TNFR1 mediated signalling leads to increased *Ripk3* expression in CX3CR1^+^ monocytes, and to an overall induction of RIPK3 activity in the chronically inflamed livers in the *Mdr2*^*−/−*^ background, which is not associated with necroptotic cell death.

### CX3CR1^+^ monocytes and TH17 cells accumulate in livers of *Tnfr1*^*−/−*^*/Mdr2*^*−/−*^ mice over time

**Figure 5:**
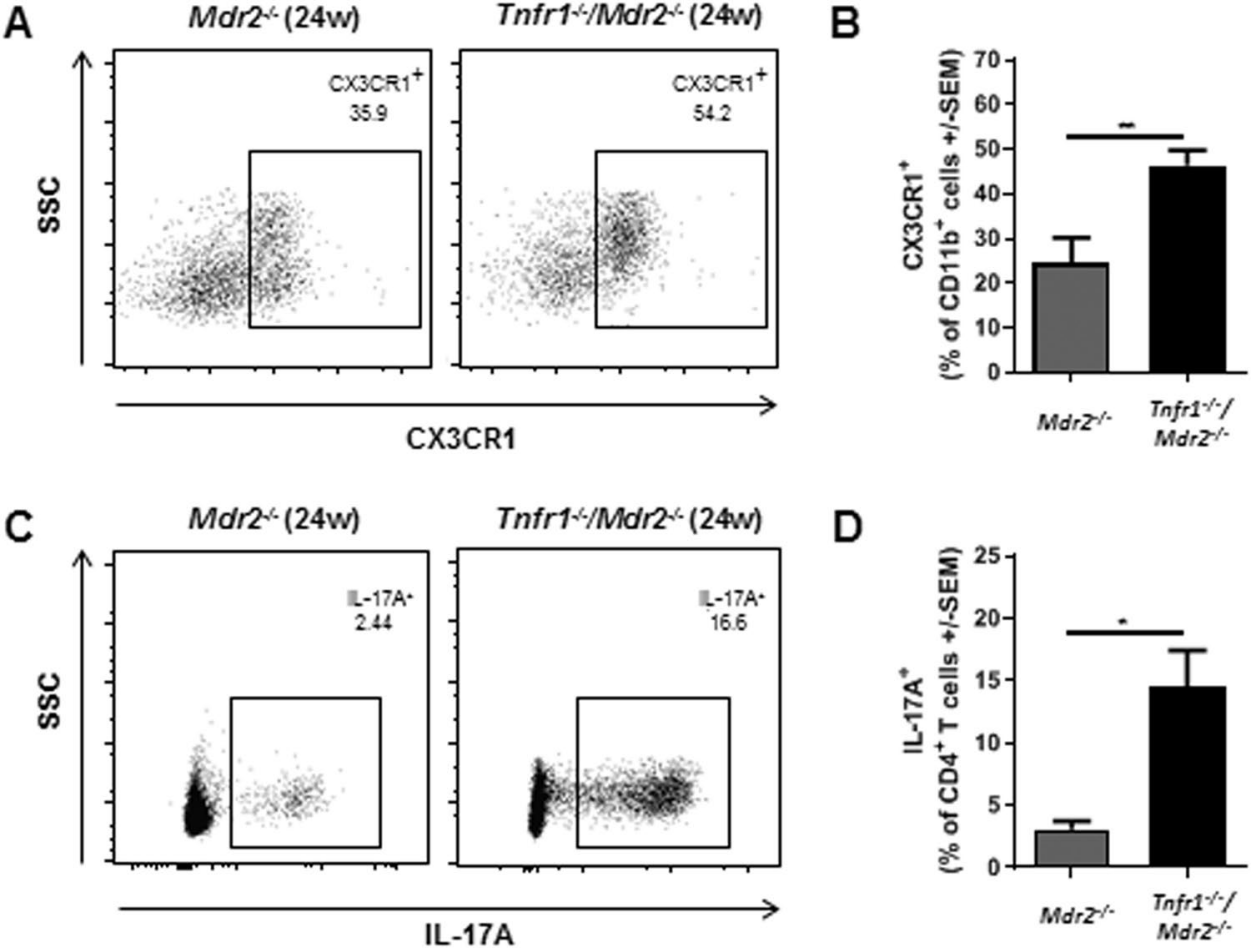
CX3CR1^+^ monocyte and TH17 cells accumulate in livers of *Tnfr1*^*−/−*^*/Mdr2*^*−/−*^ mice with disease progression. (**A**) Representative dot plots and (**B**) quantification of flow cytometric analysis of CD11b^+^CX3CR1^+^ cell populations in the livers of 24-week-old *Mdr2*^*−/−*^ (n ≥ 3), and *Tnfr1*^*−/−*^*/Mdr2*^*−/−*^ (n ≥ 6) mice determined by flow cytometry. (**C**) Representative dot plots and (**D**) quantification of flow cytometric analysis of TCRβ^+^CD4^+^IL-17^+^ TH17 cell populations in the livers of mice described in (**A**,**B**) determined by flow cytometry. **P ≤ 0.01.

While *Ripk3* expression was increased in hepatic CD11b^+^CX3CR1^+^ monocytes of 12-week-old *Tnfr1*^*−/−*^*/Mdr2*^*−/−*^ mice, we did not determine increased amounts of these cells at that age. In order to rule out time-dependent effects, we also analysed CD11b^+^CX3CR1^+^ cells in livers of 24-week-old *Mdr2*^*−/−*^ and *Tnfr1*^*−/−*^*/Mdr2*^*−/−*^ mice via flow cytometry. Here observed an increased frequency of CD11b^+^CX3CR1^+^ monocytes in livers of *Tnfr1*^*−/−*^*/Mdr2*^*−/−*^ mice compared to *Mdr2*^*−/−*^ mice (Fig. 5A,B, gating strategy in Supplementary Fig. 2C). In addition, over time the TH17 cell population equally increased in *Tnfr1*^*−/−*^*/Mdr2*^*−/−*^ mice, and much less in *Mdr2*^*−/−*^ mice (Fig. 5C,D). In summary, the differences in the hepatic immune cell composition of *Tnfr1*^*−/−*^*/Mdr2*^*−/−*^ versus *Mdr2*^*−/−*^ mice became increasingly apparent over time.

## Discussion

Chronic inflammation of the liver, mostly independent of the underlying pathology, has several major consequences including cirrhosis, liver failure and HCC development^4^. Therefore, the search for treatment options that specifically suppress pathological inflammatory processes, while retaining the physiological immune surveillance, continues. Numerous studies have shown that specific ablation of TNFR1 has beneficial effects on epithelial cell death, inflammation and fibrosis in acute and chronic hepatitis^6,7,17^. These results imply that specifically targeting of TNFR1 may be more favourable compared to targeting total TNFα-mediated signalling. However, multiple studies also showed that TNFα-mediated signalling via TNFR1 is critical for hepatocyte proliferation and regeneration^20,21^. Furthermore, preconditioning with TNFα has proven to be protective against ischemia/reperfusion injury^30^. The collective data presented in this study clearly demonstrate that the constitutive ablation of TNFR1 in a mouse model of chronic liver inflammation does not improve the pathological phenotype. Instead, elevated plasma levels of ALT and ALP combined with a higher hepatic collagen content clearly showed increased tissue injury and fibrogenesis in *Tnfr1*^*−/−*^*/Mdr2*^*−/−*^ mice compared to the *Mdr2*^*−/−*^ mice. Due to the complete absence of phospholipids in bile, tissue injury in the *Mdr2*^*−/−*^ mouse model is in part driven by progressive cholestasis and impaired cholesterol excretion^14^. Increased levels of plasma bilirubin accompanied with a decreased cholesterol output observed in *Tnfr1*^*−/−*^*/Mdr2*^*−/−*^ mice indicated a manifestation of cholestatic features in the absence of TNFR1. Subsequent analysis revealed distinct differences in the pathology of *Tnfr1*^*−/−*^*/Mdr2*^*−/−*^ and *Mdr2*^*−/−*^ mice on cellular and molecular level.

First, the divergent cytokine and chemokine profiles in livers of both mouse lines should be noted. Elevated hepatic gene expression levels of *Il-1β*, *Il-23*, *Tgfβ1*, *Il-17A*, and *Rorc* in *Tnfr1*^*−/−*^*/Mdr2*^*−/−*^ mice implied TH17 cell accumulation in the inflamed liver, which was later confirmed via flow cytometry. The roles of IL-1β and TGFβ1 in TH17 cell differentiation have been described extensively *in vitro* and *in vivo*^31,32^. IL-23 mediated signalling has been shown to be essential for stabilizing TH17 cell gene signature (*Rorγt*, *IL-17A*), down-regulation of repressive factors (*Il2, Il12*) and the induction of TH17 cell pathogenicity^33^. *Tnfr1*^*−/−*^*/Mdr2*^*−/−*^ mice showed up-regulated gene expression of several chemokines and chemokine receptors involved in TH17 cell recruitment including *Ccl2, Ccr6, and Cxcr6*^34–37^.

In addition, we observed increased hepatic expression of *Cx3cr1* in livers of *Tnfr1*^*−/−*^*/Mdr2*^*−/−*^ mice which was proportional to the increased hepatic gene expression of *Ripk3*. CX3CR1/L1 is known especially for its role in the recruitment of leucocytes including macrophages and T cell subsets^38^. While direct recruitment of TH17 cells via CX3CR1 has not been reported, TH17 cells are diminished in *Cx3cr1*^*−/−*^ mice, used in a model of collagen induced arthritis^39^, and T cell specific CX3CR1 deficiency reduced TH17 cell polarization and impaired IL-17A production *in vitro*^40^. We therefore speculate that the increased TH17 response observed in livers of *Tnfr1*^*−/−*^*/Mdr2*^*−/−*^ mice is associated with the observed increase of *Cx3cr1* gene expression and accumulation of CX3CR1^+^ monocytes over time. This assumption is supported by the fact that CX3CR1^+^ monocytes are essential for the induction of commensal-specific TH17 cells in the gut^41^, the primary site where the TH17 cell response is controlled^42^. An increasing body of evidence further emphasizes the role of the gut-liver axis in a variety of inflammatory hepatic pathologies, including primary sclerosing cholangitis (PSC), a chronic cholestatic liver disease for which *Mdr2*^*−/−*^ mice are used as a disease model^43–45^. Eighty percent of PSC patients also suffer from inflammatory bowel disease (IBD), and recent reports have shown that *Mdr2*^*−/−*^ mice display increased gut permeability and sensitivity to dysbiosis^46,47^. Interestingly our data showed that in the absence of TNFR1 CD11b^+^CX3CR1^+^ monocytes express higher levels of *Ripk3*, which is concomitant with an overall increase of hepatic RIPK3 activation in *Tnfr1*^*−/−*^*/Mdr2*^*−/−*^ mice compared to *Mdr2*^*−/−*^ mice. Previous reports showed that RIPK3 has necroptosis-independent immune modulatory functions in gut derived monocytes. Moriwaki *et al*. demonstrated that RIPK3 mediates injury-induced production of IL-1β and IL-23 in a CX3CR1^+^ monocytic population during dextran sulfate sodium induced colitis^29^. Moreover, they showed that RIPK3 is essential for initiating tissue repair via induction of IL-22. This is in line with our observation of significantly increased plasma levels of IL-22 in *Tnfr1*^*−/−*^/*Mdr2*^*−/−*^ mice and robust expression of several markers of regeneration such as *Pcna*, *Ccna2*, and *Cdk1*. This finding is in contrast to previous reports showing that TNFR1 is essential for successful initiation of liver regeneration, via the NFκB, IL-6, STAT3 axis^21^. However, TH17 cells are known to produce high levels of IL-22 whereas IL-17 has been shown to induce IL-6 production via multiple pathways including AKT and NFκB activation^48–50^. In line with that, we did not observe significantly reduced plasma levels of IL-6 in *Tnfr1*^*−/−*^*/Mdr2*^*−/−*^ mice. We therefore hypothesize that hepatic regeneration of *Tnfr1*^*−/−*^/*Mdr2*^*−/−*^ mice may be maintained by the increased numbers of TH17 cells and their production of IL-17A and IL-22 in the injured liver of *Tnfr1*^*−/−*^*/Mdr2*^*−/−*^ mice. While compensatory proliferation during chronic tissue injury is essential to retain tissue integrity, it is also the basis for tumour development^51^. It has to be noted that none of the animals used in this study showed macro- or microscopic signs of tumorous tissue, neither at 12- nor at 24-weeks of age. However, we analysed the hepatic expression of genes known to be up-regulated in HCCs and found that *Tnfr1*^*−/−*^/*Mdr2*^*−/−*^ mice expressed significantly more A20, OPN, and *Afp* than *Mdr2*^*−/−*^ mice^27,28,52^. Considering that *Tnfr1*^*−/−*^/*Mdr2*^*−/−*^ mice displayed a more severe pathology and active proliferation, we speculate that during chronic liver inflammation ablation of TNFR1 has rather a detrimental than a beneficial effect on tumour development. Overall, we conclude that the absence of TNFR1 signalling exacerbates the pathological phenotype of *Mdr2*^*−/−*^ mice, demonstrated by increased liver injury presumably due to increased IL-17A mediated signalling. Moreover, the increased disease severity in *Tnfr1*^*−/−*^/*Mdr2*^*−/−*^ mice compared to *Mdr2*^*−/−*^ is associated with a divergent cytokine and chemokine milieu which consequently leads to an altered immune cell composition enriched in TH17 cells and increased recruitment of CX3CR1^+^ monocytes over time. This study implies several interesting paths for future research, including a closer look on the role of TNFR1 on cellular and microbial homeostasis in the gut, the organ responsible for TH17 cell priming. It would further be of high interest to further elucidate the interplay of TNFR1 and RIPK3, and how targeted neutralization of one of the signalling molecules shaped immune modulatory functions of the other.

## Material and Methods

### Mice

For the phenotypical analysis of *Tnfr1*^*−/−*^/*Mdr2*^*−/−*^mice, a C57/BL6 background was chosen. The *Mdr2*^*−/−*^ (C57BL/6.129P2-Abcb4tm1Bor) mice were kindly provided by Daniel Goldenberg (Jerusalem, Israel). The *Tnfr1*^*−/−*^ (C57BL/6-Tnfrsf1atm1Imx/J) mice were kindly provided by Volker Vielhauer (Munich, Germany). The *Tnfr1*^*−/−*^/*Mdr2*^*−/−*^ mice were generated by cross-breeding of homozygous specimen of the single knockouts. Successful knockout was confirmed via PCR analysis of DNA isolated from tail biopsies. All mice received human care according to the FELASA guidelines implemented by National Institutes of Health. All mice received care according to the FELASA guidelines. The animal protocols were approved by the Hamburg Federal Authority for Health and Environment and are in accordance with the legal and ethical requirements in Germany. Mice were housed in IVC cages under controlled conditions (22 °C, 55% humidity, and 12-hour day-night rhythm) and fed a standard laboratory chow (LASvendi, Soest; Altromin, Lage, Germany).

### Determination of plasma enzymes and cytokines

Liver damage was assessed by measuring plasma enzyme activity of alanine aminotransferase (ALT) and alkaline phosphatase (ALP) as described previously^16^. Plasma levels of IL-6 and -22 were determined via Legendplex (Biolegend, San Diego, CA) according to manufacturer’s instruction.

### Hydroxyproline assay

Assays were performed as described previously^53^.

### Immunohistochemistry

Sirius Red staining was as described previous^54^. Images were taken with a BZ-9000 microscope (Keyence, Osaka, Japan). Sirius Red positive areas were quantified with BZ-II Analyzer software (Keyence, Osaka, Japan).

### Flow cytometry

Immune cell composition was determined via flow cytometry. Cells were analysed with LSRFortessa (BD bioscience, Franklin Lake, NJ). Obtained data were interpreted using FlowJo (BD bioscience, Franklin Lake, NJ) software. Antibodies are summarized in Table 1. The gating strategy for identifying the different hepatic immune cell subsets is depicted in Supplementary Fig. 2A,C.

**Table 1:** Antibodies.

	Target	Fluorophore/Host	Clone	Distributed by
**Flow cytometry**				
T cells	TCR (β chain)	Pe-Cy7	H57–597	BioLegend, San Diego, CA
	CD4	FITC	RM4–5	B BioLegend, San Diego, CA
	IL-17	Alexa Fluor 700	TC11–18H10.1	BioLegend, San Diego, CA
Monocytes	CD45	BV570	30-F11	BioLegend, San Diego, CA
	CD11b	Alexa Fluor 700	M1/70	BioLegend, San Diego, CA
	CX3CR1	BV785	SA011F11	BioLegend, San Diego, CA
**Fluorescence-activated cell sorting**				
	CD11b	PerCp-Cy5.5	M1/70	BioLegend, San Diego, CA
	CX3CR1	BV785	SA011F11	BioLegend, San Diego, CA
**Western blot**				
	RIPK3-P	goat	EPR9516(N)-25	Abcam, Cambridge, UK
	MLKL-P	goat	EPR9515(2)	BD Pharmigen, San Jose, CA
	GAPDH	goat	polyclonal	Santa Cruz, Dellas, TX

### Fluorescence-activated cell sorting

A single cell suspension of hepatic NPCs was generated using standard laboratory techniques. Cells were stained with an antibody cocktail described in Table 1B. CD11b^+^CX3CR1^+^ and CD11b^+^CX3CR1^−^ were sorted with a FACSAria III cell sorter (BD bioscience, Franklin Lake, NJ) using FACSDiva software (BD bioscience, Franklin Lake, NJ). The gating strategy is depicted in Supplementary Fig. 2B.

### *Ex vivo* re-stimulation of hepatic non-parenchymal cells NPCs and determination of cytokine production

Isolated NPCs from the liver, were re-stimulated with phorbol-12-myristate-13-acetate (PMA) (50 ng/ml) and ionomycin (1 µg/ml) for 4 h at 37 °C. Supernatant was collected and stored at −20 °C. Cytokines were quantified with Legendplex (Biolegend, San Diego, CA) according to manufacturer’s instruction. For analysis of TH17 cells via flow cytometry, brefeldin A (50 ng/ml) and monensin (1 µg/ml) were added for intracellular cytokine accumulation.

### Detection of messenger RNA by quantitative real-time reverse transcriptase polymerase chain reaction (RT-qPCR)

Isolation of total RNA, complementary DNA synthesis, and RT-qPCR were performed as described previously^16^. Oligonucleotides were obtained from Metabion International AG (Steinkirchen, Germany) and are summarized in Table 2.

**Table 2:** Oligonucleotide Sequences.

Target	Forward Primer	Reverse Primer	Reference
*Atp5b*	ATTGCCATCTTGGGTATGGA	AATGGGTCCCACCATGTAGA	NM_016774
*Il1b*	TCATGGGATGATGATGATAAC	CCCATACTTTAGGAAGACACG	NM_008361.4
*Il23*	GACTCAGCCAACTCCTCCAG	GGCACTAAGGGCTCAGTCAG	NM031252
*Tgfb1*	GAAGTGGATCCACGAGCC	CTGCACTTGCAGGAGCGC	M13177
*Il17a*	TCCAGAAGGCCCTCAGACTA	AGCATCTTCTCGACCCTGAA	U043088
*Rorc*	GAGCCAAGTTCTCAGTCATGAG	GGCCAAACTTGACAGCATCT	AAD46913
*Ccl2*	TCCCAATGAGTAGGCTGGAG	GCTGAAGACCTTAGGGCAGA	NM_011333.3
*Cxcl1*	GCTGGGATTCACCTCAAGAA	TGGGGACACCTTTTAGCATC	NM_008176.3
*Ccr6*	GTTGAACATGGCCATCACAG	CGTCAGTGTTCTGGAGCGTA	NM001190333
*Cxcr6*	TAGTGGCTGTGTTCCTGCTG	GGCAGCCGATATCCTTCATA	NM030712
*Ripk1*	CCCCGATTTGAAGAGGCTTG	CTTCGTTTCCAGCTCCTTCG	X80937
*Ripk3*	GTACTTGGACCCAGAGCTGT	CTGTCACACACTGTTTCCCG	AF178953
*Acta2*	GCATCCACGAAACCACCTAT	AGGTAGACAGCGAAGCCAAG	X13297
*Col1a1*	GAGCGGAGAGTACTGGATCG	TACTCGAACGGGAATCCATC	NM007742
*Col3a1*	GTCCACGAGGTGACAAAGGT	GATGCCCACTTGTTCCATCT	NM009930
*Mmp2*	CAGCAAGTAGATGCTGCC	CAGCAGCCCAGCCAGTC	NM008610
*Mmp9*	CATTCGCGTGGATAAGGAGT	ACCTGGTTCACCTCATGGTC	NM_013599
*Timp1*	CATCAATGCCTGCAGCTTC	CAAGCAAAGTGACGGCTC	NM011593
*Timp2*	CTCTGTGACTTCATTGTGCC	CACGCGCAAGAACCATCAC	NM011594
*Pcna*	CCACATTGGAGATGCTGTTG	CAGTGGAGTGGCTTTTGTGA	X53068
*Ccna22*	GTGGTGATTCAAAACTGCCA	AGAGTGTGAAGATGCCCTGG	NM_009828.2
*Cdk1*	GGCGACTCAGAGATTGACCA	TTGCCAGAGATTCGTTTGGC	NM_007659.3
*Tnfaip3*	CCAGGTTCCAGAACAATGTC	CTC CAT ACA GAG TTC CTC AC	U19463
*Ssp1*	CTCTGATCAGGACAACAAC	CCTCAGAAGATGAACTCTC	AF515708
*Afp*	AGCAAAGCTGCGCTCTCTAC	GAGTTCACAGGGCTTGCTTC	NM007423

### Protein isolation from mouse liver and western blot analysis

Tissue lysates were prepared as described previously^16^. Semi-quantitative evaluations were performed using VersaDoc M Imaging System, 4000 MP (Bio-Rad, Hercules, CA). Antibodies are summarized in Table 1.

### Statistical Analysis

Statistical analyses were performed using graphpad prism 7 software (GraphPad Software, La Jolla, CA). All data are presented as mean ± SEM. For comparisons between 2 groups either a students’s t-test or when applicable a non-parametric Mann-Whitney test were used. For comparisons of more than 2 groups a one-way ANOVA with Tukey’s post-hoc test was used. Correlation between 2 parameters were determined via Spearman non-parametric correlation test. Outliers were identified applying the ROUT method. *P ≤ 0.05, **P ≤ 0.01, ***P ≤ 0.001, ****P ≤ 0.0001. Asterisks above columns represent significance of the difference compared to WT.

The datasets generated during and/or analysed during the current study are available from the corresponding author on reasonable request.

## Supplementary information

Supplementary Figures

## References

[1] Byass P (2014). The global burden of liver disease: A challenge for methods and for public health. BMC Med..

[2] Goldberg DS, Fallon MB (2015). The Art and Science of Diagnosing and Treating Lung and Heart Disease Secondary to Liver Disease. Clinical Gastroenterology and Hepatology.

[3] Wong F (2015). The evolving concept of acute kidney injury in patients with cirrhosis. Nat. Rev. Gastroenterol. Hepatol..

[4] Davis GL (2008). Hepatocellular carcinoma: management of an increasingly common problem. Proc (Bayl Univ Med Cent).

[5] Pellicoro A, Ramachandran P, Iredale JP, Fallowfield JA (2014). Liver fibrosis and repair: Immune regulation of wound healing in a solid organ. Nat. Rev. Immunol..

[6] Cubero FJ (2013). TNFR1 determines progression of chronic liver injury in the IKKgamma/Nemo genetic model. Cell Death Differ.

[7] Tarrats N (2011). Critical role of tumor necrosis factor receptor 1, but not 2, in hepatic stellate cell proliferation, extracellular matrix remodeling, and liver fibrogenesis. Hepatology.

[8] MacEwan DJ (2002). TNF receptor subtype signalling: Differences and cellular consequences. Cell. Signal..

[9] Sedger LM, McDermott MF (2014). TNF and TNF-receptors: From mediators of cell death and inflammation to therapeutic giants - past, present and future. Cytokine Growth Factor Rev..

[10] Ali T (2013). Clinical use of anti-TNF therapy and increased risk of infections. *Drug Heal*. Patient Saf.

[11] Steeland S (2015). Generation and characterization of small single domain antibodies inhibiting human tumor necrosis factor receptor 1. J Biol Chem.

[12] Van Hauwermeiren F, Vandenbroucke RE, Libert C (2011). Treatment of TNF mediated diseases by selective inhibition of soluble TNF or TNFR1. Cytokine Growth Factor Rev.

[13] Smit JJM (1993). Homozygous disruption of the murine MDR2 P-glycoprotein gene leads to a complete absence of phospholipid from bile and to liver disease. Cell.

[14] Fickert P (2004). Regurgitation of bile acids from leaky bile ducts causes sclerosing cholangitis in Mdr2 (Abcb4) knockout mice. Gastroenterology.

[15] Popov Y, Patsenker E, Fickert P, Trauner M, Schuppan D (2005). Mdr2 (Abcb4)−/− mice spontaneously develop severe biliary fibrosis via massive dysregulation of pro- and antifibrogenic genes. J Hepatol.

[16] Barikbin R (2012). Induction of heme oxygenase 1 prevents progression of liver fibrosis in Mdr2 knockout mice. Hepatology.

[17] Shibata H (2008). The therapeutic effect of TNFR1-selective antagonistic mutant TNF-alpha in murine hepatitis models. Cytokine.

[18] Leist M (1996). The 55-kD tumor necrosis factor receptor and CD95 independently signal murine hepatocyte apoptosis and subsequent liver failure. Mol. Med..

[19] Koerber K, Sass G, Kiemer AK, Vollmar AM, Tiegs G (2002). *In vivo* regulation of inducible NO synthase in immune-mediated liver injury in mice. Hepatology.

[20] Yamada Y, Fausto N (1998). Deficient liver regeneration after carbon tetrachloride injury in mice lacking type 1 but not type 2 tumor necrosis factor receptor. Am J Pathol.

[21] Yamada Y, Kirillova I, Peschon JJ, Fausto N (1997). Initiation of liver growth by tumor necrosis factor: deficient liver regeneration in mice lacking type I tumor necrosis factor receptor. Proc Natl Acad Sci USA.

[22] van Nieuwerk CM (1997). The role of bile salt composition in liver pathology of mdr2 (−/−) mice: differences between males and females. J Hepatol.

[23] Aggarwal BB, Gupta SC, Kim JH (2012). Historical perspectives on tumor necrosis factor and its superfamily: 25 years later, a golden journey. Blood.

[24] Brockmann L, Giannou AD, Gagliani N, Huber S (2017). Regulation of TH17 cells and associated cytokines in wound healing, tissue regeneration, and carcinogenesis. Int. J. Mol. Sci..

[25] Katzenellenbogen M (2007). Molecular Mechanisms of Liver Carcinogenesis in the Mdr2-Knockout Mice. Mol. Cancer Res..

[26] Asadzadeh Z (2017). The paradox of Th17 cell functions in tumor immunity. Cell. Immunol..

[27] Dong B, Lv G, Wang Q, Wang G (2012). Targeting A20 enhances TRAIL-induced apoptosis in hepatocellular carcinoma cells. Biochem. Biophys. Res. Commun..

[28] Cao L (2015). Osteopontin promotes a cancer stem cell-like phenotype in hepatocellular carcinoma cells via an integrin–NF-κB–HIF-1α pathway. Oncotarget.

[29] Moriwaki K, Balaji S, Bertin J, Gough PJ, Chan FK-M (2017). Distinct Kinase-Independent Role of RIPK3 in CD11c(+) Mononuclear Phagocytes in Cytokine-Induced Tissue Repair. Cell reports.

[30] Teoh N, Leclercq I, Pena AD, Farrell G (2003). Low-dose TNF-alpha protects against hepatic ischemia-reperfusion injury in mice: implications for preconditioning. Hepatology.

[31] Chung Y (2009). Critical regulation of early Th17 cell differentiation by interleukin-1 signaling. Immunity.

[32] Hatton RD (2011). TGF-b in Th17 cell development: the truth is out there. Immunity.

[33] Gaffen SL, Jain R, Garg AV, Cua DJ (2014). The IL-23-IL-17 immune axis: from mechanisms to therapeutic testing. Nat. Rev. Immunol..

[34] Su X (2010). Tumor Microenvironments Direct the Recruitment and Expansion of Human Th17 Cells. J. Immunol..

[35] Turner J-E (2010). CCR6 Recruits Regulatory T Cells and Th17 Cells to the Kidney in Glomerulonephritis. Journal of the American Society of Nephrology: JASN.

[36] Hirota K (2007). Preferential recruitment of CCR6-expressing Th17 cells to inflamed joints via CCL20 in rheumatoid arthritis and its animal model. J. Exp. Med..

[37] Butcher MJ, Wu CI, Waseem T, Galkina EV (2016). CXCR6 regulates the recruitment of pro-inflammatory IL-17A-producing T cells into atherosclerotic aortas. Int. Immunol..

[38] Lee M, Lee Y, Song J, Lee J, Chang S-Y (2018). Tissue-specific Role of CX3CR1 Expressing Immune Cells and Their Relationships with Human Disease. Immune Netw..

[39] Tarrant TK (2012). Decreased Th17 and antigen-specific humoral responses in CX3CR1-deficient mice in the collagen-induced arthritis model. Arthritis Rheum..

[40] Dong L (2016). T Cell CX3CR1 Mediates Excess Atherosclerotic Inflammation in Renal Impairment. J. Am. Soc. Nephrol..

[41] Panea, C. *et al*. Intestinal Monocyte-Derived Macrophages Control Article Intestinal Monocyte-Derived Macrophages Control Commensal-Specific Th17 Responses. 1314–1324 10.1016/j.celrep.2015.07.040 (2015). 10.1016/j.celrep.2015.07.040 PMC4567384 26279572

[42] Esplugues E (2011). Control of T H 17 cells occurs in the small intestine. Nature.

[43] Palmela C, Peerani F, Castaneda D, Torres J, Itzkowitz SH (2017). Inflammatory Bowel Disease and Primary Sclerosing Cholangitis: A Review of the Phenotype and Associated Specific Features. Gut Liver.

[44] Patman G (2015). CX3CR1 - A direct line to gut-liver crosstalk?. Nat. Rev. Gastroenterol. Hepatol..

[45] Eksteen B (2016). The Gut-Liver Axis in Primary Sclerosing Cholangitis. Clin. Liver Dis..

[46] Tedesco D (2018). Alterations in Intestinal Microbiota Lead to Production of Interleukin 17 by Intrahepatic γδ T-cell Receptor-positive Cells and Pathogenesis of Cholestatic Liver Disease. Gastroenterology.

[47] Eaton JE (2013). Pathogenesis of primary sclerosing cholangitis and advances in diagnosis and management. Gastroenterology.

[48] Liang SC (2006). Interleukin (IL)-22 and IL-17 are coexpressed by Th17 cells and cooperatively enhance expression of antimicrobial peptides. J. Exp. Med..

[49] Gu F-M (2011). IL-17 induces AKT-dependent IL-6/JAK2/STAT3 activation and tumor progression in hepatocellular carcinoma. Mol. Cancer.

[50] Chen Y, Kijlstra A, Chen Y, Yang P (2011). IL-17A stimulates the production of inflammatory mediators via Erk1/2, p38 MAPK, PI3K/Akt, and NF-κB pathways in ARPE-19 cells. Mol. Vis..

[51] Karin M (2006). Nuclear factor-κB in cancer development and progression. Nature.

[52] Li, G.-C. Tumor markers for hepatocellular carcinoma (Review). *Mol. Clin. Oncol*. 593–598 10.3892/mco.2013.119 (2013). 10.3892/mco.2013.119 PMC3915636 24649215

[53] Uchinami H, Seki E, Brenner DA, D’Armiento J (2006). Loss of MMP 13 attenuates murine hepatic injury and fibrosis during cholestasis. Hepatology.

[54] Segnani C (2015). Histochemical Detection of Collagen Fibers by Sirius Red/Fast Green Is More Sensitive than van Gieson or Sirius Red Alone in Normal and Inflamed Rat Colon. PLoS One.

