# Supplementary material for: Deletion of tumour necrosis factor α receptor 1 elicits an increased TH17 immune response in the chronically inflamed liver
Source: Sci Rep. 2019 Mar 12;9:4232. doi: 10.1038/s41598-019-40324-z (PMC6414655; doi:10.1038/s41598-019-40324-z)
Supplement: Supplementary file 1 — Supplementary Figures [file 41598_2019_40324_MOESM1_ESM.pdf]

Deletion of tumour necrosis factor α receptor 1 elicits an increased TH17 immune response in the chronically inflamed liver

Laura Berkhout, Roja Barikbin, Birgit Schiller, Gevitha Ravichandran, Till Krech, Katrin Neumann, Gabriele Sass, Gisa Tiegs

Supplementary Figures

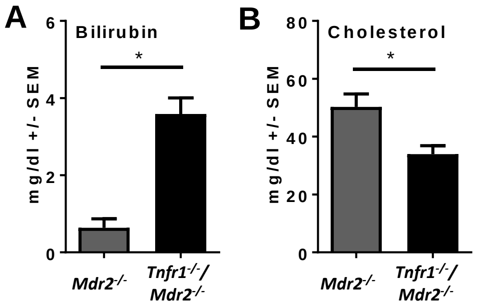

Supplementary Figure 1: Marker of cholestasis in *Tnfr1$^{-/-}$/Mdr2$^{-/-}$* mice.
(A) Plasma concentrations of bilirubin and
(B) cholesterol in *Mdr2$^{-/-}$* (n = 4) and *Tnfr1$^{-/-}$/Mdr2$^{-/-}$* (n = 5) mice (10-12 weeks of age). *P ≤ 0.05

A <u>Gating strategy: flow cytometric anaylsis of TH17 cells (Figure 3)</u>

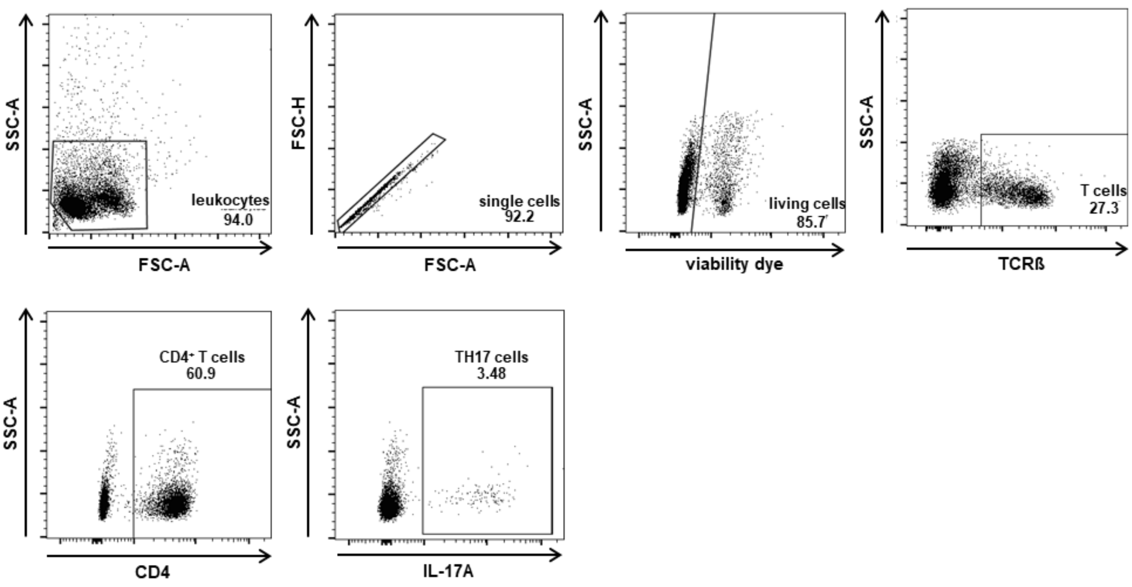

B <u>Gating strategy: fluorescence-activated cell sort of CD11b$^+$CX3CR1$^{-/+}$ cells (12w; Figure 4)</u>

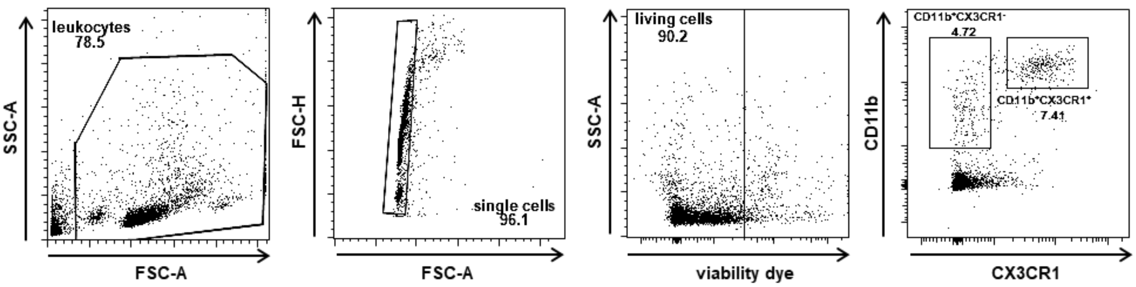

C <u>Gating strategy: flow cytometric analysis of CD11b$^+$CX3CR1$^+$ cells (24w; Figure 5)</u>

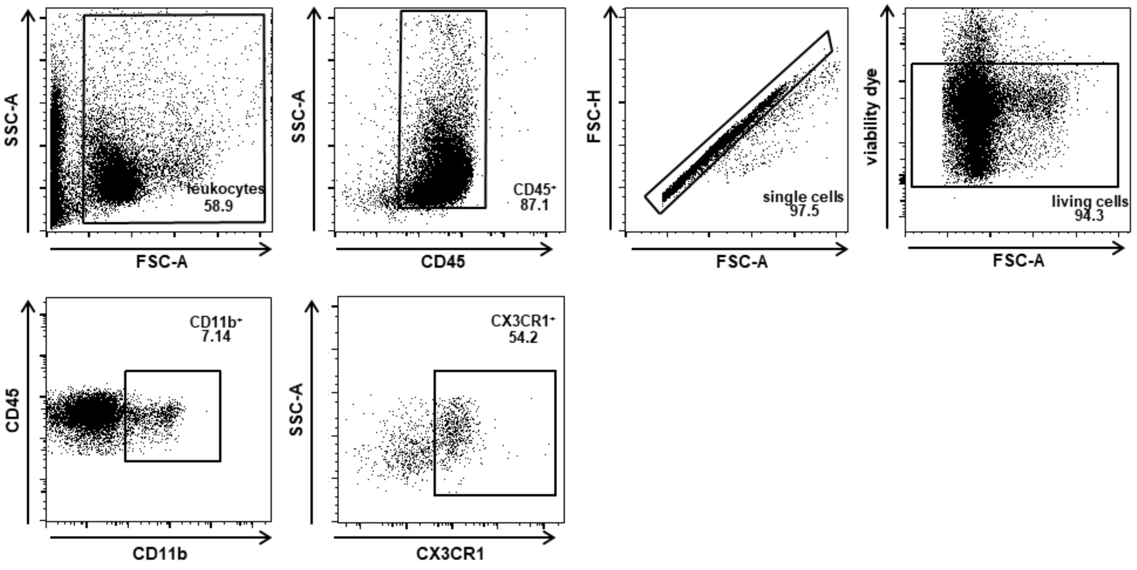

Supplementary Figure 2: Gating strategies.

For all analysis living total leukocytes were determined by forward / sideward scatter, and exclusion of dead cells. (A) TH17 cells were defined as TCRß$^+$ CD4$^+$ IL-17$^+$ cells. (B) Cells were sorted for CD11b$^+$ and CX3CR1$^{+/-}$. (C) Cells were defined as CD45$^+$ CD11b$^+$ and CX3CR1$^+$.

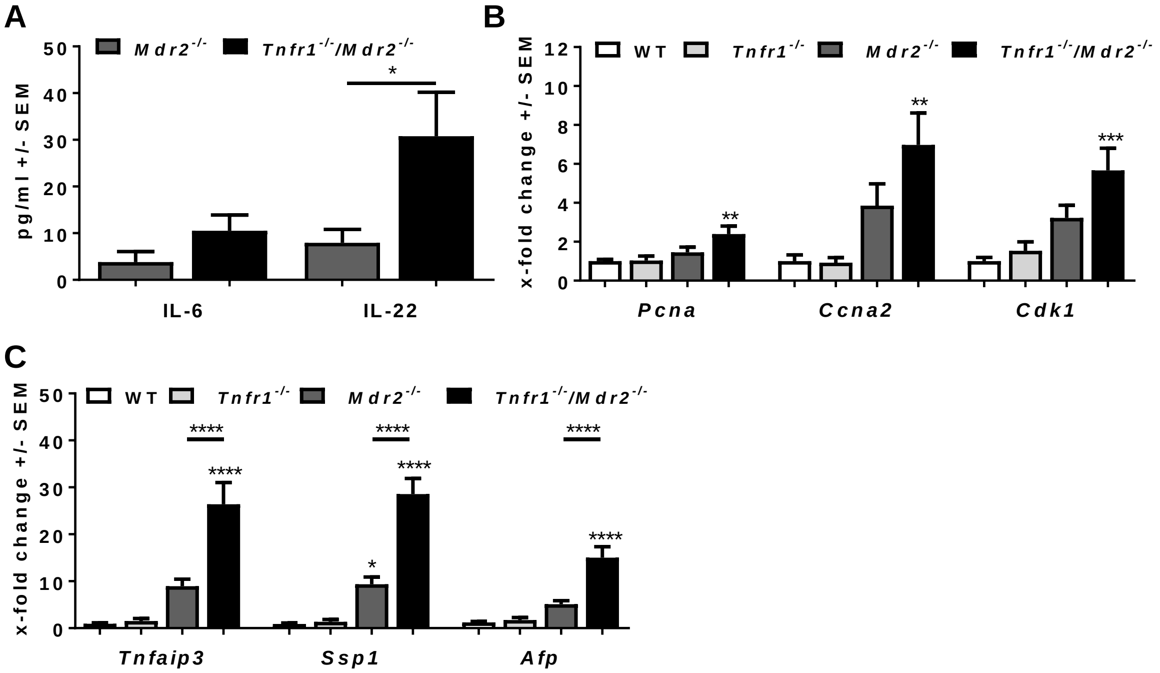

Supplementary Figure 3: Hepatic proliferation and differentiation in *Tnfr1*$^{-/-}$/*Mdr2*$^{-/-}$ mice. (A) Plasma levels of STAT3 inducing cytokines IL-6 and IL-22 in *Mdr2*$^{-/-}$ (n = 8) and *Tnfr1*$^{-/-}$/*Mdr2*$^{-/-}$ (n = 8) mice measured via Legendplex. (B) Hepatic gene expression analysis of proliferation marker *Pcna*, *CcnA2* and *Cdk1* and (C) tumour marker *Tnfaip3*, *Ssp1*, and *Afp* in WT (n ≥ 10), *Tnfr1*$^{-/-}$ (n = 9), *Mdr2*$^{-/-}$ (n ≥ 9) and *Tnfr1*$^{-/-}$/*Mdr2*$^{-/-}$ (n ≥ 10) determined by RT-qPCR. *P ≤ 0.05, **P ≤ 0.01, ***P ≤ 0.001.

Full length western blots pf phosphorylated RIP3, MLKL and GAPDH as loading control

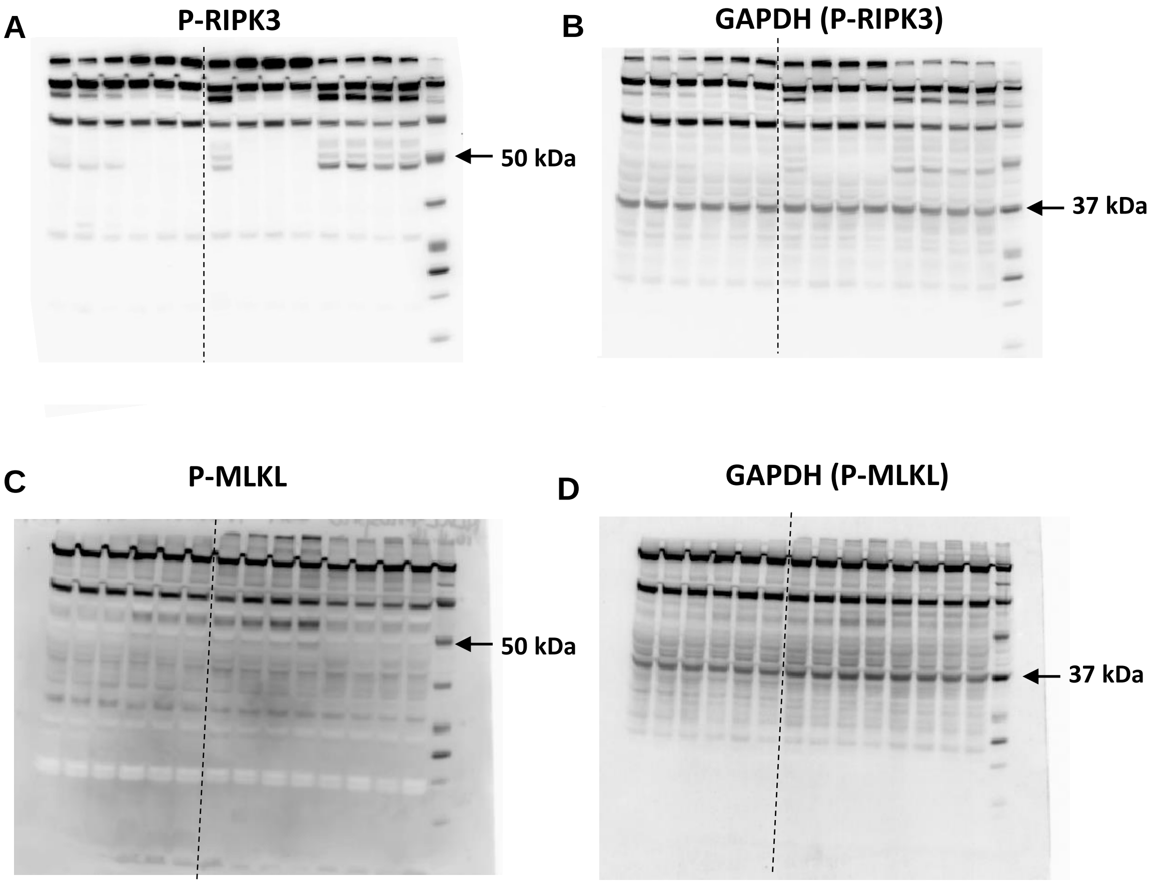

Supplementary Figure 4: Full length western blots of data presented in Figure 4B.
The liver tissue samples lysates for the western blot analysis of P-RIPK3 and P-MLKL were derived from the same experiment and gels/blots were processed in parallel. Gels were loaded as follows, left lane 1-3 WT, Lane 4-6: *Tnfr1*$^{-/-}$, lane 7-10: *Mdr2*$^{-/-}$, lane 11-14: *Tnfr1*$^{-/-}$/*Mdr2*$^{-/-}$, Lane 15: molecular marker (Precision Plus Protein WesternC Standards). Each lane depicts one animal. Only lanes 7-15 are displayed in the main article, dashed lines indicate were images were cropped.
